# Emerging Treatments and Therapies for Autism Spectrum Disorder: A Narrative Review

**DOI:** 10.7759/cureus.63671

**Published:** 2024-07-02

**Authors:** Alan D Kaye, Kaitlyn E Allen, Van S Smith III, Victoria T Tong, Vivian E Mire, Huy Nguyen, Zachary Lee, Maria Kouri, Carlo Jean Baptiste, Chizoba N Mosieri, Adam M Kaye, Giustino Varrassi, Sahar Shekoohi

**Affiliations:** 1 Department of Anesthesiology, Louisiana State University Health Sciences Center, Shreveport, USA; 2 School of Medicine, Louisiana State University Health New Orleans School of Medicine, New Orleans, USA; 3 School of Medicine, Louisiana State University Health Sciences Center, Shreveport, USA; 4 Anesthesia, National and Kapodistrian University of Athens, Athens, GRC; 5 Department of Pharmacy Practice, Thomas J. Long School of Pharmacy, University of the Pacific, Stockton, USA; 6 Pain Medicine, Paolo Procacci Foundation, Rome, ITA

**Keywords:** music therapy, comorbidities, complementary, oxytocin, autism spectrum disorder

## Abstract

The prevalence of autism spectrum disorder (ASD) has increased over the last decade. In this regard, many emerging therapies have been described as ASD therapies. Although ASD does not have a cure, there are several management options available that can help reduce symptom severity. ASD is highly variable and, therefore, standard treatment protocols and studies are challenging to perform. Many of these therapies also address comorbidities for which patients with ASD have an increased risk. These concurrent diagnoses can include psychiatric and neurological disorders, including attention deficit and hyperactivity disorder, anxiety disorders, and epilepsy, as well as gastrointestinal symptoms such as chronic constipation and diarrhea. Both the extensive list of ASD-associated disorders and adverse effects from commonly prescribed medications for patients with ASD can impact presenting symptomatology. It is important to keep these potential interactions in mind when considering additional drug treatments or complementary therapies. This review addresses current literature involving novel pharmacological treatments such as oxytocin, bumetanide, acetylcholinesterase inhibitors, and memantine. It also discusses additional therapies such as diet intervention, acupuncture, music therapy, melatonin, and the use of technology to aid education. Notably, several of these therapies require more long-term research to determine efficacy in specific ASD groups within this patient population.

## Introduction and background

When the Diagnostic and Statistical Manual of Mental Disorders, Fifth Edition (DSM-5) was updated in 2013, the diagnostic criteria and classifications for pervasive developmental disorders (PDD) were changed to autism spectrum disorders (ASD). The new naming system was chosen to better describe the wide variety of symptoms and concurrent disorders that are often seen in patients with ASD [1]. Although there is a vast spectrum of features and severity, there are still characterizable symptoms associated with ASD, including difficulty with social interactions, repetitive behavioral patterns, and reduced adaptive capabilities. These symptoms present early in childhood, usually by 24 months old, and disrupt daily functions [2]. The etiology of ASD is not currently known, and the age of onset is also variable. However, the prevalence has increased substantially to 1:68, according to the Centre for Disease Control National Center on Birth Defects and Developmental Disabilities' fiscal year 2014 annual report, from 1:500 less than a decade before [1,3]. One potential reason for this change is the increase in copy number variations and several gene variations in patients [1,2]. It is important to note that although criteria changes reflected in the DSM-5 did occur amid the increasing prevalence, reports show a 17% decrease in ASD prevalence when moving from the DSM-4 PDD criteria to the DSM-5 criteria [4].

Because ASD is highly variable, the assessment and treatment protocols involve a multi-disciplinary approach. ASD screening is recommended for all children at nine, 18, 24, or 30 months old to assess risk. If screening indicates some related symptoms are present, then a series of diagnostic interviews are completed. The presence of concurrent disorders should also be investigated since there is a higher correlation between ASD and diagnoses such as cognitive or language impairment, attention deficit and hyperactivity disorder (ADHD), or epilepsy [1]. Early intervention is highly important for the best outcomes, and treatment options can include chronic behavioral, psychosocial, pharmacological, and complementary therapies, which cater to the individual’s specific symptomatology [1].

Because there is an abundance of non-evidence-based treatment options available for ASD, there is a concern that the marketing of these treatments can take advantage of patients and families. As these individuals comprise a vulnerable population, it is important to increase awareness and promote research to distinguish evidence-based practices from the rest. One study's survey of parents of children with ASD reported a total of 111 distinct treatments tried, emphasizing the quantity of these options available [5]. Because many non-evidence-based practices are costly and may cause harm to patients, practitioners must be aware of what options have evidentiary support [1]. For this reason, therefore, the present investigation includes a critical evaluation of novel treatments for ASD as well as current standards of care, comorbidities, and newer pharmacological and non-pharmacological therapies identified in the world literature.

## Review

Current standard of care

Although there is no cure for ASD, the current standard of care focuses on early diagnosis and symptom-based intervention. In a meta-analysis by Vivanti et al., the importance of early diagnosis is highlighted by significantly improved developmental and social outcomes in patients diagnosed between ages two and five years [6,7]. The American Association for Child and Adolescent Psychiatry (AACAP) recommends using the Modified Checklist for Autism in Toddlers (M-CHAT) for screening, followed by further diagnostic evaluations for symptomatic patients. Clinical evaluation should also investigate comorbid conditions that are frequently associated with ASD [1,8]. There are no reliable diagnostic biomarkers for this disorder, so family history, physical examinations, and observable behavioral characteristics based on the DSM-5 criteria are vital factors in diagnosis [8-10].

Current interventions for ASD are highly variable and include both non-pharmacological and pharmacological treatments. Non-pharmacological therapies typically include educational interventions focusing on academics, socialization, adaptation, and communication. These specific educational strategies include applied behavior analysis (ABA), the TEACCH (Treatment and education of autistic and related communication handicapped children) method, developmental methods, speech and language therapy, and sensory integration therapy [11].

In 1987, the Lovaas method of ABA yielded significant increases in IQ scores and educational function of patients with ASD. In the original study, 47% of the ABA treatment group achieved normal educational function and normal-range IQ score, while only 2% of the control group achieved this outcome [12,13]. Lovaas’ method incorporates intense, long-term, individualized behavioral interventions to accommodate the developmental process of young patients [12,13]. It has since been modified, but it still serves as the foundation for current behavioral interventions [12]. Several subsequent types of ABA, such as discrete trial training (DTT), early intensive behavioral interventions (EIBI), pivotal response training (PRT), and verbal behavioral intervention (VBI), each work through re-conditioning target behaviors [8].

Structured teaching via the TEACCH method aims to challenge students by tailoring instruction to their deficits, improving skills, and providing social interaction. It allows students to learn at their own pace while alleviating common challenges that often impact these patients. This opposes the standard school system, which expects students to keep up with the preset pace regardless of learning disabilities [11]. Developmental models can also be used to assist with skills related to emotional regulation and social communication. Some developmental models include the Denver model, early start Denver model (ESDM), developmental individual difference (DIR), relationship developmental intervention (RDI), and responsive teaching (RT) [8].

Patients with ASD often present with neurological challenges that impact sensory and motor functions. Many patients experience language deficits, including repetitive speech, narrow interest, uneven language development, or lack of speech [10]. These patients can be referred to speech-language pathologists for assistance with general social communication [11]. For delays in motor development, atypical motor coordination, and difficulty with learned muscle movement and control, physical therapy is another useful resource [14]. Additionally, children with ASD can frequently become overwhelmed and exhibit sensory over-responsivity, making it challenging to filter relevant stimuli and focus on strenuous tasks [15]. Occupational therapists can use sensory integration therapy (SIT) to promote adaptive processing of sensory stimuli. This therapy teaches patients to perform daily tasks tied to self-care and social interaction while integrating a better understanding of their environment via their senses [11,15]. Since these non-pharmacological methods are highly associated with each other, components of each method are typically utilized together, along with medications, to form a specialized therapeutic regimen based on patient deficits and goals [11].

Serotonin is an important mediator in the proper functioning of the GI tract, central nervous system, and cardiovascular system, and studies have demonstrated elevated peripheral serotonin levels in ASD [12,16]. It is generally believed that this elevation results in the loss of serotonin terminals during development, which may also be affecting serotonin terminals in the brain [17]. Therefore, serotonin regulators such as selective serotonin reuptake inhibitors (SSRIs), serotonin-norepinephrine reuptake inhibitors (SNRIs), and tricyclic antidepressants (TCAs) have been prescribed to help relieve symptoms of anxiety and certain repeated behaviors [12]. Irritability is another key symptom of ASD and is a target for atypical antipsychotics, such as risperidone and aripiprazole [12]. Other standard pharmacological treatments correlate with concurrent disorders present. These may include stimulants for ADHD or melatonin for sleep disturbances [12]. Generally, the mainstay treatment for ASD includes both educational and pharmacological interventions, and novel treatments are continuously being developed with promising results.

Comorbidities associated with ASD

Not only does ASD extensively vary in presentation, but these individuals also experience a higher burden of somatic and psychiatric comorbid disorders. Estimates report as many as 74% of patients present with at least five other concurrent disorders [18]. The management of these symptoms includes pharmacologic agents that often overlap and interact with one another, and the rate of polypharmacy seen in this patient population is between 6.8% and 87% [19]. The neurobiological interactions between the accompanying disorders and ASD require comprehensive assessment approaches, modified cognitive behavioral therapy, and carefully managed pharmacological interventions to avoid the risks of polypharmacy and ensure effective care.

Patients with ASD have a higher burden of genetic disorders such as Fragile X syndrome, Down syndrome, and Duchenne muscular dystrophy [20-23]. Several categories of psychiatric disorders such as ADHD (28%), anxiety disorders (20%), sleep disorders (13%), and depressive disorders (11%) are also comorbid with ASD [24-28]. These disorders can confound the presenting behavioral and developmental symptoms, resulting in atypical findings and complicated diagnoses. Neurological comorbidities such as cerebral palsy, macrocephaly, and epilepsy have been observed, and the risk of epilepsy can be partly predicted by ASD severity [20,29]. These patients are commonly prescribed psychotropic medications including antipsychotics, antidepressants, benzodiazepines, hypnotics, and stimulants. It can also be challenging to detect adverse effects in patients with ASD related to difficulty reporting associated with intellectual disabilities and the overlap of comorbid symptoms [30].

Children with ASD present with GI symptoms early in development. GI dysfunction is associated with the severity of psychiatric symptoms and negative clinical outcomes [31]. Further research reveals a bi-directional nature between gut dysbiosis and systemic immune dysregulation, and current literature suggests it is likely that untreated GI disorders can directly contribute to cognitive and behavioral deficits [32]. GI symptoms are amongst the most prevalent comorbidities found in ASD, occurring at a rate that is three times higher than their typical peers [33]. Some commonly reported disorders are chronic constipation, diarrhea, gastroesophageal reflux, and inflammatory bowel disease. Many of the medications used to treat comorbid psychiatric disorders, such as stimulants, affect bowel function and can cause or worsen these GI symptoms [20].

Comorbidities can exacerbate ASD symptoms as well as complicate the management of the disorder. Recently, the rate of medical intervention and polypharmacy has risen to address various symptoms of the diseases. Polypharmacy is a major concern for clinicians as these drugs can have serious adverse effects and various drug interactions. Over time, many patients frequently change their drug regimens while attempting to find the best balance between symptom improvement and adverse effects [34]. Possible drivers for pharmaceutical transiency can include patient preference, cost considerations, adverse effects, or treatment efficacy. There are limited studies on the concomitant various pharmacological agents and behavioral therapy as treatment options for ASD. More research is required to ensure effective care by optimizing the current understanding of ASD and its comorbidities, providing better diagnostic tools, and implementing clinical guidelines for treatment.

Novel pharmacological treatments

There are many established pharmacologic therapies proven to help with comorbidities related to ASD, such as atypical antipsychotics for aggression and irritability, SSRIs or SNRIs for anxiety, depression, and obsessive-compulsive disorder, and methylphenidate for ADHD [35]. There are also many emerging targeted drug therapies with promising study results, including oxytocin, bumetanide, acetylcholinesterase inhibitors, and memantine [12,35]. This section will focus on these new therapies and their potential efficacy in treating symptoms of ASD.

Oxytocin

Oxytocin is a neuropeptide produced in the paraventricular and supraoptic nuclei of the hypothalamus and released by the posterior pituitary, and it plays a role in social functioning. Studies in animals and normal developing adults have demonstrated enhanced social processing following oxytocin administration [36-39]. Given that ASD is characterized by social impairments, it has been theorized that oxytocin therapy can improve these deficits. Guastella et al. performed a double-blind, crossover, placebo-controlled study with 16 males aged 12-19 diagnosed with ASD, treated with intranasal oxytocin, and evaluated with the Reading the Mind in the Eyes task. Improved emotion recognition was seen in the oxytocin administration group, even at lower doses [40]. Gordon and colleagues performed a study on 17 children with ASD, measuring changes in brain activity while looking at socially and non-socially meaningful images following oxytocin administration. Using functional magnetic resonance imaging (fMRI), they found that oxytocin enhanced brain activity in children for socially meaningful stimuli [41]. More recently, a meta-analysis by Huang et al. in 2021 found that oxytocin led to improvements in social functioning and some core ASD symptoms [42]. However, the systematic review by Iffland and colleagues in 2023 suggests that although oxytocin may reduce irritability, there is very low certainty associated with its beneficial effects [43].

Bumetanide

Bumetanide is a loop diuretic that inhibits sodium-potassium-chloride co-transporters. In the brain, it results in reduced serum chloride levels, which are linked to gamma-aminobutyric acid (GABA)-ergic inhibition and improved symptoms of ASD [44]. Sixty children aged 3-11 years were evaluated in a randomized controlled clinical trial and received bumetanide or placebo for three months. Researchers found that bumetanide significantly reduced the Childhood Autism Rating Scale and the Clinical Global Impressions [44]. Another randomized controlled study on 91 children with ASD demonstrated improvement in repetitive behaviors but did not have any significant improvement in core ASD symptoms with bumetanide [45]. Two small randomized controlled trials also demonstrated improvement in ASD symptomatology after three months of bumetanide treatment [46,47]. All studies noted that bumetanide has minimal side effects aside from a risk of mild hypokalemia. Since the data presented were collected in smaller studies, results are inconclusive as to whether or not bumetanide is an effective treatment for core ASD symptoms.

Acetylcholinesterase Inhibitors

Acetylcholine has a role in memory and attention, and low levels have been explored as a potential contributor to ASD [48]. Rivastigmine, donepezil, and galantamine are all acetylcholinesterase inhibitors with slightly different mechanisms of action. Rivastigmine is currently used in the treatment of dementia [35]. It is thought to increase cholinergic activity and has been shown to improve cognition in patients with Alzheimer’s disease [49]. An open-label study was conducted for 12 weeks on 32 individuals with ASD, demonstrating significant improvement in expressive speech and overall autistic behaviors [49]. A randomized controlled trial compared donepezil to placebo in 34 individuals with ASD aged 8-17. The study did not find any significant differences in autism symptoms between the two groups [48]. Another randomized controlled trial compared galantamine plus risperidone to risperidone treatment alone in 40 individuals aged 4-12. There were no significant differences found between groups aside from a small decrease in irritability in the galantamine plus risperidone group [48]. Overall, the evidence for acetylcholinesterase inhibitor treatment for ASD is inconclusive and requires further exploration.

Memantine

The N-methyl-D-aspartate (NMDA) receptor antagonist, memantine, is also used as a treatment for Alzheimer's disease, which is linked to continuous activation of NMDA [35]. The use of memantine to treat ASD is being explored due to evidence of abnormalities in glutamate metabolism seen in imaging, genetic, and post-mortem studies [50]. A double-blind, placebo-controlled study examined the effects of memantine treatment in forty children for ten weeks. Significant improvement was seen on the Aberrant Behavior Checklist-Community in the subscales of irritability, stereotypical behavior, and hyperactivity [51]. A retrospective study demonstrated significant improvement in social withdrawal and inattention in 18 individuals with PDD treated with memantine [52]. Chez et al. performed an open-label study where 151 individuals with an ASD or PDD diagnosis were given memantine for 21 months. Researchers found significant improvement in language function, social, and self-stimulatory behaviors [53]. In 2022, Brignell and colleagues completed a systematic review and found no significant difference between memantine and placebo for any primary or secondary outcomes associated with ASD with low certainty. However, the authors noted that the studies included in the analysis had a high risk of bias, so the effects of memantine should continue to be studied in larger longitudinal studies [54].

Although current literature for these novel pharmaceutical treatments of ASD has revealed conflicting data on their effects on ASD, it is important to note that these studies also reflect the highly heterogeneous nature of this disorder. For this reason, future research should be conducted to review the effectiveness of these treatment options stratified to varying severities of ASD.

Novel complementary and alternative therapies

Alongside novel developments in pharmacological research for ASD, studies have also focused on advances in complementary therapies. Some of these alternative methods include dietary interventions, acupuncture, music therapy, melatonin, and advances in technology.

Dietary Intervention

Several dietary interventions have been studied in association with ASD, including elimination diet, ketogenic diet, and other supplements. An elimination diet raises concern due to potential nutrient deficiency. For instance, dairy products are eliminated in a casein-free diet, subsequently decreasing calcium intake, which is crucial for bone and tooth health [55]. The strict ketogenic diet is characterized by high fat, low carbohydrate, and adequate protein levels. This diet is often supplemented by various vitamins, as well [56]. Several studies suggest that a ketogenic diet in children and adolescents has effectively reduced seizure frequency in refractory epilepsy, which can be associated with ASD [56-58]. Comparing ketogenic and gluten casein (GFCF) diets, a case-control study by El-Rashidy et al. reported improvements in the Childhood Autism Rating Scale (CARS) and Autism Treatment Evaluation Test (ATEC) scales among individuals with ASD after six months in both diet groups. However, the ketogenic diet yielded more significant improvements over the GFCF diet in the subgroups of cognition and sociability [59]. To note, overconsumed vitamins or supplements can cause unintentional harm and side effects to patients without medical consultation. For example, omega-3, zinc, and iron can cause GI problems. Additionally, high levels of iron can cause iron poisoning, and high vitamin A consumption can be hepatotoxic [60]. Although many patients with ASD have nutritional insufficiencies, it is important to counsel patients and families on the side effect profiles of supplements and make recommendations based on specific deficiencies.

Acupuncture

Studies about acupuncture have been limited since many do not use a truly blinded methodology. However, in two studies examined by DeFilippis in 2018, sham acupuncture was introduced as a placebo [61]. This method targets points a few millimeters away from established acupoints. The first study reviewed electro-acupuncture and used standard-of-care measures such as the Clinical Global Impression-Improvement (CGI-I) [62], the Functional Independence Measure for Children (WeeFIM®) [63], and the Pediatric Evaluation of Disability Inventory (PEDI) as primary outcomes [64]. Electro-acupuncture is described as a portable device that conducts an adjustable wave frequency of 5-100 Hz while keeping the density of wave frequency five times the spacing wave frequency [65]. Significant improvements were noted in all three categories in the treatment group [61,66]. The second randomized controlled trial reviewed tongue acupuncture in 50 children aged 3-11 years. Results showed significant improvements in the self-care and cognition sections of the WeeFIM for those receiving active treatment [61,66]. According to the systematic review by Li et al. in 2023, high-quality evidence on acupuncture for ASD is lacking, but initial studies indicate that future research is warranted [67]. Additionally, results from Cheuk et al. suggested that acupuncture was not effective in relieving core ASD symptoms [68]. The improvements shown in some secondary measures, such as communication and global functioning, were evident in individual studies but not in the pooled data, indicating the potential benefit of large stratified studies.

Music Therapy

Impairment of social interpersonal skills is a characteristic symptom of ASD, and music therapy could have promising therapeutic effects. Functional imaging has shown that music can modulate brain activity in crucial emotion-processing areas, such as the amygdala, cingulate cortex, hippocampus, hypothalamus, insula, nucleus accumbens, and orbitofrontal cortex [69]. Studies have also reported that patients with ASD can recognize and process the emotional aspects of music, as indicated by fMRI activation of brain regions notably deficient with other emotional stimuli [69,70]. A meta-analysis of over 600 patients with ASD by Ke et al. in 2022 concluded that music therapy resulted in significant improvements in social interaction within a convenient and short therapy session [71]. However, longitudinal studies still need to be conducted to discern its long-lasting effects. Another systematic review in 2022 including over 1100 participants reported with moderate certainty that music therapy can help with short and medium-term global functioning and overall severity of ASD symptoms [72].

Others

Sleep disturbances leading to increased irritability might contribute to a decreased efficacy of ABA [73]. Initial non-pharmacological intervention can be effective, but melatonin can be used in refractory cases of insomnia for long-term maintenance with high efficacy [74]. The use of technology can also augment education and integrate learning with computer-programmed platforms [75]. The systematic review by Valencia et al. emphasizes the utility of technology to make education more accessible and engaging through gamification and notes that the predictable structure of computers can assist patients with ASD in easily keeping their routines [75].

Each of the studies on these complementary therapies could increase validity and reliability through longer study periods and more defined patient parameters. Although much of the evidence supporting these options is mixed, it is still possible that some novel complementary therapies are effective in certain patients. Articles from the last five years discussing the specific mechanisms of action and indications for each ASD-related medication, standard and novel, are compiled in Table 1.

**Table 1:** Mechanism of action, indications, and considerations of pharmacological interventions associated with ASD ASD: autism spectrum disorders, SSRIs: selective serotonin reuptake inhibitors, SNRIs: serotonin-norepinephrine reuptake inhibitors, TCAs tricyclic antidepressants, DAT: dopamine transporters, NET: norepinephrine transporters, ADHD: attention deficit and hyperactivity disorder, KCC2: K-Cl co-transporter, NMDA: N-methyl-D-aspartate

Drug or Class	Mechanism of Action	Indications	Considerations
Risperidone (atypical antipsychotic) [76]	Dopamine D2, serotonin 5-HT2, and α2 receptor antagonist	Treatment of irritability and potential reduction of repetitive behaviors, agitation, aggression, atypical motor movements, hyperactivity, and impulsivity associated with ASD.	Common adverse effects include increased appetite, weight gain, risk of metabolic syndrome, hyperprolactinemia, and acute extrapyramidal symptoms. FDA-approved for children over 5.
Aripiprazole (atypical antipsychotic) [76]	Dopamine D2 antagonist or partial agonist, serotonin 5-HT1A and 5-HT2C partial agonist, 5HT2A antagonist, and weak antagonist at H1, M1, and α1-adrenergic receptors.	Effective in short- and long-term maintenance of irritability, hyperactivity, and stereotypical behaviors associated with ASD.	FDA approved for patients ages 5-16 years. Common adverse effects include somnolence, increased appetite, and weight gain (less prominent than in risperidone).
Serotonin modulators (SSRIs, SNRIs, and TCAs) [12]	Block the reuptake of serotonin alone (SSRIs) or with norepinephrine (SNRIs and TCAs) in the synaptic cleft.	Anxiety, irritability, and mood disorders associated with ASD.	Studies are inconsistent when reporting the benefits of serotonergic medications on core ASD symptoms and may depend on the genetic subtype of ASD (ex., idiopathic vs Fragile X Syndrome associated ASD).
Methylphenidate [77,78]	Blocks dopamine transporters (DAT) in the striatum, leading to increased extracellular dopamine. Blocks norepinephrine transporters (NET) in the prefrontal cortex, leading to increased extracellular norepinephrine.	Hyperactivity and inattention due to ADHD associated with ASD.	Adverse effects include decreased appetite and sleep disturbances. Used as a first-line treatment for moderate to severe ADHD in patients age 6 and older.
Melatonin [79]	Activates high-affinity ML1 receptors and low-affinity ML2 receptors, which regulates the circadian rhythm.	Improve sleep onset latency and total sleep duration and efficiency.	It is most effective in patients who also use behavioral interventions. Adverse effects can include drowsiness, hypothermia, headache, and rash.
Intranasal Oxytocin [80]	The MOA in relation to ASD has not been fully elucidated.	May enhance social functioning and emotional recognition.	Most efficacious dose, frequency, and route of administration are still unknown.
Bumetanide [81]	NKCC1 transporter antagonist. KCC2 (K-Cl co-transporter) is expressed more than NKCC1 in the normal brain, causing higher extracellular concentrations of Cl-. When GABA-A channels open, Cl- enters and hyperpolarizes the neuron. In patients with ASD, this gradient is flipped, with NKCC1 being more highly expressed. Therefore, when GABA-A channels open, Cl- is released, and the neuron depolarizes, leading to a hyperexcitable brain. Bumetanide is thought to modulate this imbalance.	May improve recognition of facial emotions, reduce ASD-related behaviors, and increase communication abilities.	Studies show the dose for maximum efficacy is 1 mg twice per day. The dose for maximum safety is 0.5 mg twice per day. The most common adverse effects were mild hypokalemia, dehydration, and muscle weakness.
Acetylcholinesterase inhibitors (donepezil, galantamine, and rivastigmine) [48]	Inhibit acetylcholinesterase, which increases acetylcholine (ACh) levels. ACh plays a role in attention, novelty seeking, and memory, and low levels have been found in ASD.	It is not currently indicated, but more studies are needed. No conclusive evidence has suggested acetylcholinesterase inhibitors are effective in treating core ASD symptoms, including social interaction, communication, irritability, or ASD-related behaviors.	Some adverse effects may include nervousness, drowsiness, increased appetite, and tremor.
Memantine [81]	Noncompetitive and low-affinity NMDA (N-methyl-D-aspartate) receptor antagonist.	Potentially beneficial for social interaction and may assist with irritability, stereotypical behaviors, and hyperactivity when added to risperidone. It may also affect memory, communication, and cognition in ASD.	Studies have been contradictory regarding efficacy, but the drug was highly tolerable among patients. Memantine is currently used for patients with Alzheimer's who exhibit continuous activation of NMDA.

Discussion

As the prevalence of ASD has increased substantially over the past decade, the need for novel therapies to address ASD-associated symptoms and comorbidities has also grown. This increasing prevalence and the lack of a cure leave this patient population and their families as a markedly vulnerable population for claims of efficacious treatment options that lack evidentiary support. These advertised treatments can be very costly and may ultimately cause harm to these patients and families [1]. However, there are also several potential treatment options for ASD that have not yet shown conclusive evidence in the current literature but may be effective in individual cases due to the heterogeneity of ASD. Therefore, it is imperative to promote research in these areas in hopes of more conclusive recommendations regarding novel treatments and therapies.

In summary, continued research is needed for ASD treatment options and should reflect the heterogeneity of the patient population. Future directions should focus on potential biomarkers to aid in the diagnosis and stratification of patients. Without relying solely on observable behaviors, standardized biomarkers could allow researchers to study various treatment options and their potential benefits with a more precise understanding of their mechanisms of action [82]. This could also help elucidate the inconclusive findings of currently researched therapeutic options and reveal if these treatments may be more beneficial for certain subgroups of patients with ASD. Although biomarker research is also new, initial results have been promising and will hopefully continue to shape the future of ASD research and treatment into the future.

## Conclusions

In the present investigation, the standard of care for ASD was summarized, including emphasis on early diagnosis and personalized treatment plans, according to the symptomatology of the patient. This care also considers the many comorbidities that are more often seen in patients with ASD compared to their neurotypical counterparts. Because concurrent diagnoses of intellectual disabilities, ADHD, GI disorders, sleep disturbances, and many others are frequent, polypharmacy is common and must be carefully monitored.

As novel pharmacological treatments were addressed, namely oxytocin, bumetanide, acetylcholinesterase inhibitors, and memantine, the major takeaway is that more conclusive evidence is needed on efficacy and long-term benefits before making any official recommendations. Other complementary methods such as music therapy and the addition of melatonin to other behavioral therapies have proven to be beneficial for ASD symptom severity and associated sleep disorders, respectively. Still, several therapies such as acupuncture and diet interventions should be monitored carefully to ensure no unnecessary harm or discomfort is brought on to the patient for inconclusive benefits.
